# Inverted Toe-Nails

**Published:** 1882-12-20

**Authors:** 


INVERTED TOE-NAILS.—A new, simple and effectual method of operating for that troublesome little customer—ingrowing toe-nail—will be reported in the next issue of our Journal.

